# Botulinum toxin A for the Treatment of Androgenetic Alopecia: A Review

**DOI:** 10.7759/cureus.111513

**Published:** 2026-06-25

**Authors:** Laith Basch, Caden Carver, Randy Jacobs

**Affiliations:** 1 Dermatology, Arizona College of Osteopathic Medicine, Midwestern University, Glendale, USA; 2 Dermatology, Omni Dermatology, Inc., Glendale, USA; 3 Dermatology, University of California Riverside (UCR) School of Medicine, Riverside, USA

**Keywords:** androgenetic alopecia, botulinum toxin a, bta, hair regrowth, male pattern baldness

## Abstract

Androgenetic alopecia (AGA) is a condition that predisposes patients to patterned hair loss across the scalp, significantly impacting self-esteem and mental health. Conventional treatments, including finasteride, a 5-alpha-reductase, and minoxidil, a topical vasodilator, have both shown inconsistent effectiveness and are associated with notable adverse effects. Botulinum toxin type A (BTA), a neurotoxin produced by the bacterium *Clostridium botulinum*, is widely used in cosmetic medicine for reducing facial wrinkles and is indicated for several medical conditions. It has been proposed that BTA may benefit patients with AGA by increasing scalp blood flow and reducing scalp tension, though these mechanisms remain under investigation. This narrative review evaluates the current literature on BTA and its effects on androgenetic alopecia, with emphasis on efficacy, safety, and use in combination with finasteride and minoxidil. A literature search of PubMed, Medline, and Cumulative Index to Nursing and Allied Health Literature (CINAHL) Plus using the terms (Androgenetic alopecia) AND (Botulinum toxin) AND (Treatment) identified 86 articles, which were narrowed to 56 after initial screening; six of these were selected for inclusion based on their relevance to the review's objectives. All six articles reported that BTA was associated with increased hair growth as a standalone therapy and in combination with finasteride and minoxidil, with improvements in hair density, reduction in total hair loss area, and a favorable adverse effect profile. While BTA use for AGA remains in the early phases of investigation, the available evidence suggests a positive association between BTA and hair regrowth in AGA. Further research is needed to fully establish its role in the management of this condition.

## Introduction and background

Androgenetic alopecia (AGA) is a progressive condition affecting nearly 50% of both male and female individuals, characterized by patterned hair thinning and loss driven by genetic predisposition [1]. Beyond its physical manifestations, AGA significantly impacts mental health and self-confidence. Standard treatments include finasteride, a 5-alpha-reductase inhibitor, and minoxidil, a topical vasodilator, both of which have improved hair quality in many patients but are associated with inconsistent efficacy and notable adverse effects [2,3].

Botulinum toxin type A (BTA) is a neurotoxin produced by the bacterium *Clostridium botulinum*. BTA inhibits acetylcholine release, causing localized muscle paralysis [4]. Widely used in cosmetic medicine for reducing facial wrinkles, BTA is also indicated for chronic migraine, spastic disorders, cervical dystonia, and detrusor hyperactivity [4]. More recently, BTA has shown promising results in hair regrowth by reducing scalp tension, increasing local blood flow, and potentially downregulating transforming growth factor beta (TGF-β) [5]. BTA injections into the scalp paralyze the underlying musculature, reducing tension and increasing blood flow to hair follicles, which may deliver more nutrients and oxygen, potentially reversing follicular miniaturization [5]. The pathogenesis of AGA involves the conversion of testosterone to dihydrotestosterone (DHT), which drives follicular miniaturization [1]. This process is accelerated under hypoxic conditions; improved scalp oxygenation resulting from BTA-induced vasodilation may therefore attenuate DHT-driven hair loss [6].

In addition to its vascular effects, BTA-induced muscle paralysis reduces mechanical strain on hair follicles, potentially decreasing miniaturization [5,7]. DHT upregulates TGF-β, a cytokine that inhibits follicular cell proliferation and promotes miniaturization. BTA has been shown to suppress TGF-β secretion, potentially halting this process, though further clinical validation is required [7].

Collectively, these proposed mechanisms, such as reduced scalp tension, enhanced blood flow, and suppressed TGF-β activity, position BTA as a candidate therapy for AGA. Preliminary evidence indicates that BTA may improve outcomes both as a standalone treatment and in combination with finasteride and minoxidil [8]. The aim of this review is to analyze the available literature on BTA for the treatment of AGA, with emphasis on efficacy, safety, and combination therapy.

## Review

Methods

A comprehensive literature search was carried out across PubMed, Medline, and Cumulative Index to Nursing and Allied Health Literature (CINAHL) Plus using the search terms (Androgenetic alopecia) AND (Botulinum toxin) AND (Treatment). Full-text clinical articles published between 2013 and 2024 were included. Review articles and publications not pertaining to the specified keywords were excluded. Duplicate records across databases were removed prior to screening. This review is presented as a narrative review; a formal meta-analysis was not conducted due to heterogeneity in study designs, dosing protocols, outcome measures, and follow-up durations across the included studies. A formal risk-of-bias assessment was not performed; however, study quality and methodological characteristics are described narratively within the Results section.

Results

**Figure 1:**
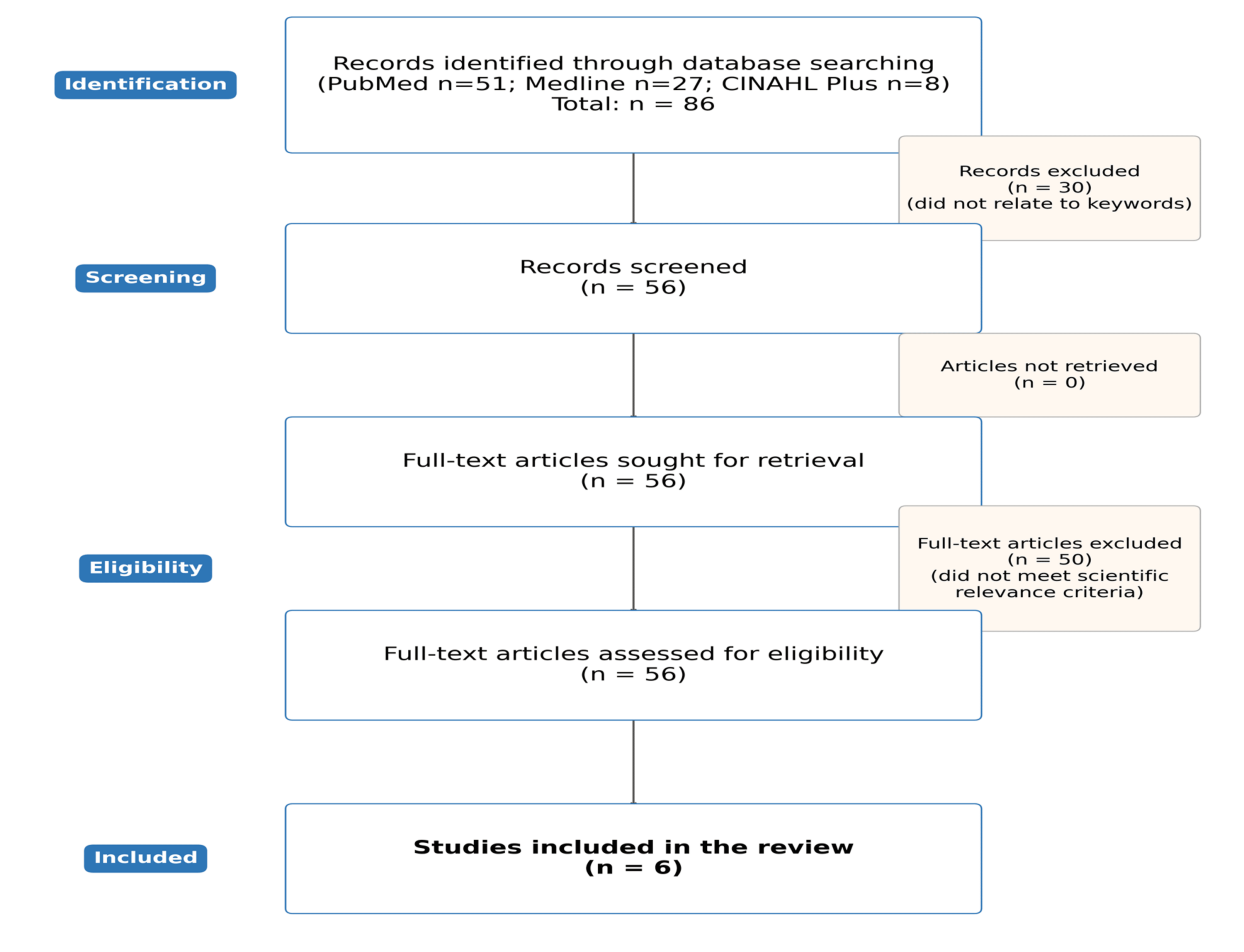
Flow diagram outlining the process of article identification and screening prior to inclusion in the review.

The initial screening found 86 articles from PubMed (n=51), Medline (n=27), and CINAHL Plus with Full Text (n=8). Articles that did not directly relate to BTA treatment for AGA or did not meet the quality criteria were excluded, resulting in 56 publications passing this initial screening. In our second screening, we used Rayyan.AI [9] to organize and further evaluate the articles; we conducted a more rigorous assessment focusing on the quality of study designs, outcome measures, and relevance to BTA treatment for AGA. Each review focused on analyzing the methods, results, and discussion. Articles that did not meet scientific relevance were excluded. This secondary screening process narrowed down the selection to six full-text publications (Figure 1).

The characteristics, methods, and key findings of the six studies included in this review are summarized in Table 1.

**Table 1:** Pertinent details of the six studies included in the review, including study type, purpose, methods, and key findings. AGA: androgenetic alopecia; BTA: botulinum toxin type A; FNS: finasteride; BTX: botulinum toxin; DHT: dihydrotestosterone; DPCs: dermal papilla cells (DPCs); AE: adverse events; TGF-β1: transforming growth factor beta 1

Author	Study Type	Purpose	Methods	Key Findings/Results
Zhou et al. [10]	Randomized Controlled Trial	To assess the effectiveness and safety of Botulinum Toxin A in treating AGA	A total of 63 patients with AGA were randomized to receive BTA alone (n = 30) or BTA combined with oral finasteride 1 mg/day (BTA+FNS; n = 33). Each session delivered 100 U of BTA across 30 scalp injection sites, repeated every 3 months for four sessions. Hair counts and standardized head photographs were assessed over 12 months.	Hair counts increased significantly in both groups throughout the treatment. The BTA+FNS group had significantly more counts of hair four sessions after treatment compared to those of the BTA group (234.01 ± 27.35 vs. 218.26 ± 30.59 root/cm^2^; p < 0.05). Hair density improved, and hair loss area reduced after each treatment. Effective rates were 73.3% for BTA and 84.8% for BTA+FNS after four sessions
Tian et al. [8]	Prospective Cohort Study	To investigate the efficacy of Botulinum Toxin A treatment in treating AGA	A total of 37 male patients with AGA. Oral finasteride (1 mg/day) and topical 5% minoxidil applied daily for six months vs. unilateral subcutaneous BTA injections (100 units, diluted with 2.5 ml of 0.9% sterile normal saline) + standard therapy. Treatment effects were assessed at three and six months. Hair density measured using Canfield's system. Skin analysis conducted using CHOWIS HAIR diagnostic tools (Chowis Co., LTD., Yongin, South Korea).	Combined unilateral BTA skin injections with standard treatment (finasteride and minoxidil) proved to be effective and increased hair density among patients with AGA. At six months, hair density was 75.7% more effective than that in the control group. Participants also identified the relief in symptoms such as oily scalp, pruritus, and dandruff. No significant adverse reactions were observed, aside from minor pain during injection.
Singh et al. [11]	Pilot Study	To evaluate the effectiveness of Botulinum Toxin in treating AGA	Ten male patients in the age group of 22-42 years. Every patient was administered a 5 U dose of BTA in 30 intramuscular injections on the scalp. Assessment was performed using photography and self-assessment scoring by patients before and after treatment.	At 24 weeks, eight out of 10 patients had a good to excellent response to treatment based on photographic assessment. One patient had a poor response, while the other had a fair one. Self-assessment showed good to excellent response in seven patients, fair in two patients, and poor in one patient. No side effects of treatment were seen during the study period.
Seoudy et al. [12]	Observational Study	To assess efficacy of different Botulinum Toxin A concentrations in treating AGA	A total of 32 AGA patients received intradermal BTA injections at two different concentrations, followed by six-month follow-ups. Patients were divided by gender. Injections were administered with concentrations of BTA 33.3 U/mL and 25 U/mL. The Ludwig and Norwood-Hamilton scales were assessed. Dermoscopy for hair density	After six months, significant improvements were observed in both male and female patients. The proportion of male patients classified as Hamilton–Norwood Grade II increased from 0% to 60%, female patients classified as Ludwig Grade I increased from 14.8% to 70%. Dermoscopic evaluation showed increase in hair density on the right side (33.3 U/mL), with fewer adverse effects reported. The left side (25 U/mL) showed less improvement in hair density and dermoscopic findings. AEs were mild and transient.
Shon et al. [5]	Mechanistic Study	To investigate the effect of intradermal Botulinum Toxin in treating AGA	A total of 18 male AGA patients were treated every four weeks with intradermal injections of BTA for 24 weeks. The BTA injections were given over the balding scalp at 20 sites with a dose of 30 units of BTA. Excluded patients taking finasteride or minoxidil. BTA action on inhibiting the secretion of TGF-β1 was tested in cultured DPCs in the presence of DHT with selected doses of BTA according to a previous study.	After 24 weeks, the mean number of hairs per square centimeter had increased from 129.61 to 136.22 (P = 0.012). Improvement was seen in the pretreatment and posttreatment photos (P = 0.031). Ex vivo, BTA-treated the DHT-induced DPCs and showed down-regulation of TGF-β1. No serious AE.
Zhang et al. [13]	Clinical Trial	To evaluate efficacy of low-dose Botulinum Toxin A in treating AGA	Five male AGA outpatients aged 30-45 years. A total of 50 units of botox into the muscles of the head, with no fewer than 30 injection sites. Efficacy and safety were assessed at baseline, three months, and six months after treatment. Scalp grease was measured using the German Derma-Expert MC760, and hair counts were also determined at each visit in the course of the treatment.	24/25 patients completed the trial. After three months, nine subjects had marked regrowth of hair (greater than 10% increase over the baseline number), 10 showed minimal growth of hair (0–10% increase from baseline number), and five continued to lose hair. 19 cases demonstrated a substantial reduction in scalp grease secretion reaching the minimum level at three months. After six months, 11 subjects had marked hair regrowth, poor improvement in eight, and no response in five. Same 19 cases showed a significant decrease, and the secretion of grease gradually returned toward normal values. No AE.

Patient Demographics

Of the six studies, the majority of AGA patients were male, though some studies included female participants. Zhang et al. [13] studied patients aged 30-45 years, while Singh et al. [11] enrolled patients aged 22-42. Seoudy et al. included both male and female patients and stratified outcomes by sex [12]. The included studies drew from varied geographic populations: Tian et al. [8] and Zhou et al. [10] conducted their studies in China, Shon et al. [5] conducted their study in Korea, and Zhang et al. [13] specifically reported from Chinese ethnicity. Ethnicities were not formally documented in the remaining studies. This narrow geographic and sex distribution limits the generalizability of findings to broader populations.

Treatment Frequency

Zhou et al. administered 100 U of BTA across 30 scalp sites every three months for four sessions, comparing BTA alone with BTA plus oral finasteride [10]. Tian et al. used 100 units of BTA via subcutaneous injection once every three months unilaterally in conjunction with conventional treatments (finasteride and minoxidil) [8]. Singh et al. used 30 intramuscular injections of five units of BTA on the scalp every six months [11]. Seoudy et al. used different concentrations of BTA on AGA patients [12]. Shon et al. used intradermal injection of 30 units of botulinum toxin every four weeks for 24 weeks [5]. Zhang et al. used 50 units of BTA injected into head muscles at no less than 30 injection sites every six months [13].

Treatment Efficacy

Several reports showed the effectiveness of BTA in AGA alone or in combination with standard treatments like finasteride and minoxidil (Table 1). In a randomized controlled trial, Zhou et al. randomized 63 patients with AGA [10]. After four treatment sessions, hair count in the BTA+FNS group was significantly greater than in the BTA group (234.01 ± 27.35 vs. 218.26 ± 30.59 root/cm², p < 0.05), as assessed by phototrichogram. After every treatment, hair density increased, and the area of hair loss decreased. Following four sessions, BTA was 73.3% effective while BTA+FNS was 84.8% effective.

Tian et al. enrolled 37 male AGA patients in a prospective cohort [8]. BTA injections combined with standard treatments were associated with a 75.7% improvement in hair density compared to the control group at six months, as measured using Canfield’s system and CHOWIS HAIR diagnostic tools (Chowis Co., LTD., Yongin, South Korea). Subjects experienced decreased scalp oiliness, pruritus, and dandruff.

Singh et al. enrolled 10 male patients with AGA; at 24 weeks, eight out of 10 patients reported good to excellent hair regrowth by photographic assessment, with no side effects during the study follow-up [11]. Seoudy et al. tested different BTA concentrations on 32 patients with AGA using dermoscopy, the Ludwig scale, and the Norwood-Hamilton scale [12]. They found that increasing the concentration to 33.3 U/mL every three months produced greater hair density improvement than 25 U/mL. Shon et al. reported a significant increase in hair counts and a decrease in TGF-β1 secretion in 18 male patients treated with BTA [5]. Zhang et al. noted hair regrowth within six months in most patients, assessed via the Derma-Expert MC760 device (Courage + Khazaka electronic GmbH, Cologne, Germany [13].

Objective measurement modalities used across the studies included phototrichogram [10], Canfield’s system [8], CHOWIS HAIR analysis [8], dermoscopy [12], photographic assessment [11], Derma-Expert MC760 [13], Ludwig scale [12], and Norwood-Hamilton scale [12]. Subjective assessments included self-assessment scoring [13], patient satisfaction surveys [8], symptom relief reporting [8], visual analog scale (VAS) [13], patient diaries [13], and quality of life (QoL) questionnaires. The heterogeneity of these outcome measures across studies limits direct comparison of efficacy results.

Adverse Event Profile

BTA was overall well-tolerated across the six studies, with adverse effects that were consistently mild, transient, and localized to the injection site. The most frequently reported adverse effect was minor pain during or shortly after injection; Tian et al. noted no significant adverse reactions aside from this injection-site discomfort [8]. Seoudy et al. observed only mild and transient effects across the concentrations tested and, notably, reported fewer adverse effects at the higher BTA concentration (33.3 U/mL) than at the lower concentration (25 U/mL), indicating that increased dosing did not raise toxicity within the ranges studied [12]. Zhang et al. [13], Zhou et al. [10], Shon et al. [5], and Singh et al. [11] each reported no adverse events attributable to BTA over their respective follow-up periods, and no serious or systemic adverse events were reported in any study. This favorable short-term safety profile likely reflects the localized nature of intradermal and subcutaneous BTA delivery and contrasts with the systemic adverse effects associated with oral finasteride. However, none of the included studies applied a standardized adverse-event grading system, and the relatively short follow-up periods limit conclusions regarding long-term safety.

Discussion

This review analyzes the literature selected to assess the use of BTA for treating male pattern baldness or AGA. The results and data collected during this review suggest that BTA may be an effective therapy for AGA, whether used alone or in conjunction with other traditional therapies, though the level of evidence remains limited. The difference between the traditional therapies and BTA was that the traditional therapies varied in their effectiveness and showed unwanted adverse effects, whereas BTA alone showed similar effectiveness and very minimal adverse effects. Mild, transient effects, such as slight pain at the site of injection and occasional headaches, were reported. Otherwise, there were no significant adverse effects [11]. It was also found that among all the literature that was analyzed, BTA results and effectiveness stayed consistent. A previously published systematic review by English and Ruiz [14] also supports the efficacy of BTA in improving hair density and reducing hair loss areas in AGA patients. The proposed mechanisms of BTA action, such as increasing scalp blood flow and reducing scalp tension, may address physiologic principles not targeted by current pharmacological treatments. As this is a narrative review without formal meta-analysis, findings are reported descriptively; heterogeneity in dosing protocols, study designs, and outcome measures across the included studies precluded pooled statistical analysis.

Minoxidil, one of the most widely used conventional therapies for male pattern baldness, is a topical treatment that vasodilates blood vessels in the scalp and increases hair growth. The disadvantage of this therapy is that it is inconsistent in how it works depending on the patient’s sulfotransferase activity [15], and it often has to be continued long-term to maintain results [6]. Finasteride, on the other hand, is a 5-alpha-reductase inhibitor and works to decrease the conversion of testosterone to DHT, the main hormone that causes hair loss in AGA. The potential disadvantages of finasteride include the adverse effects of sexual dysfunction, with some users experiencing persistent sexual side effects even after discontinuing the drug [16]. A meta-analysis by Chen et al. combined finasteride with minoxidil in treating AGA to test the efficacy and concluded that the combination has higher efficacy than either drug alone, though side effects may limit long-term adherence [17]. A separate randomized, assessor-blinded pilot trial by Rossi and Caro similarly found combination topical finasteride and minoxidil more effective than either monotherapy [18].

Overall, the included studies suggest that BTA may increase hair density and reduce hair loss area both as a standalone therapy and in conjunction with finasteride and minoxidil. In one prospective cohort, combining BTA with finasteride and minoxidil was associated with a 75.7% improvement in hair density compared to the control group [8]; however, these findings should be interpreted with caution given the small number of included studies, variable methodologies, and the absence of a formal meta-analysis. Direct comparison across studies is further limited by variability in BTA dosing, injection techniques, follow-up durations, and outcome assessment tools, reinforcing the need for standardized methodology in future trials.

These findings point toward the fact that integrating BTA into existing treatments can enhance outcomes. English and Ruiz also align with these findings, showing a synergistic effect of BTA when used with conventional AGA therapies [14]. The adverse effects of minoxidil and finasteride are well known [2]. Minoxidil’s adverse effects include irritation and allergic reactions resulting in contact dermatitis on the scalp [3]. When minoxidil is applied to the scalp, it is absorbed into the local circulation. Finasteride's adverse effects are much more systemic, which include sexual dysfunction and depression [2]. This is due to finasteride being oral and minoxidil being topical. Sexual dysfunction and depression could significantly worsen a patient’s quality of life. In contrast, BTA is highly localized to an application site, thus lowering the potential for systemic adverse effects.

When it comes to the treatment of AGA, long-term efficacy is significant because AGA is a lifelong condition. Patient compliance is paramount in all therapies and can be the limiting factor in the efficacy of hair growth. In both minoxidil and finasteride, efficacy is directly correlated with continuous use. Once the therapies are stopped, the hair loss will progress. With BTA, more long-term studies are still needed to determine the optimal treatment frequency and duration of its effects. These long-term studies also need to have larger, more randomized, and controlled trials to be able to find an ideal dose.

Furthermore, future studies need to be more diverse in demographics to investigate the effects of BTA on different ages, ethnicities, and genders. English and Ruiz also note a lack of diversity in study demographics, making it important to research across various demographics to understand BTA’s effects better [14].

Limitations

This review has several limitations that should be acknowledged. First, the number of included studies was small (n = 6), and most studies featured limited sample sizes, reducing statistical power and the generalizability of findings. Second, the included studies had relatively short follow-up periods, making it difficult to assess the long-term efficacy and durability of BTA treatment for AGA. Third, considerable heterogeneity existed across study designs, ranging from randomized controlled trials to pilot studies and observational studies, as well as in BTA dosing protocols, injection techniques, and outcome measures, which limits direct comparison. Fourth, the patient populations were predominantly male, restricting conclusions about BTA’s efficacy in female-pattern AGA. Fifth, geographic and ethnic representation was narrow, with most studies conducted in East Asian populations (China and Korea), which may limit the applicability of findings to other demographic groups. Additionally, the absence of a formal risk-of-bias assessment limits the ability to evaluate the internal validity of individual study findings. Publication bias is also a potential concern, as studies with positive outcomes may be more likely to be published, potentially overestimating BTA's therapeutic efficacy. Future research should employ larger randomized controlled trials with standardized protocols, longer follow-up periods, formal risk-of-bias assessment, and more demographically diverse populations to better characterize the efficacy and safety profile of BTA for AGA.

## Conclusions

BTA is a promising potential therapy for individuals with AGA, including those who have not responded to conventional treatments. Early evidence suggests that BTA may enhance patient outcomes and satisfaction, but its role in hair regrowth modalities remains to be established and will require further clinical investigation. Nonetheless, AGA remains a multifactorial condition that likely benefits from a multimodal therapeutic approach; the integration of BTA into established treatment regimens represents a promising avenue toward a more comprehensive management strategy for affected patients.
